# The effect of acupuncture on oxidative stress: A systematic review and meta-analysis of animal models

**DOI:** 10.1371/journal.pone.0271098

**Published:** 2022-09-09

**Authors:** Yu Zhao, Bo Zhou, Guangyin Zhang, Shixin Xu, Jipeng Yang, Shizhe Deng, Zengmin Yao, Qiang Geng, Bin Ouyang, Tian Xia

**Affiliations:** 1 Andrology Department, First Teaching Hospital of Tianjin University of Traditional Chinese Medicine, Tianjin, China; 2 National Clinical Research Center for Chinese Medicine Acupuncture and Moxibustion, Tianjin, China; 3 Psychosomatic Medicine Department, First Teaching Hospital of Tianjin University of Traditional Chinese Medicine, Tianjin, China; 4 Tianjin Key Laboratory of Traditional Research of Traditional Chinese Medicine Prescription and Syndrome, First Teaching Hospital of Tianjin University of Traditional Chinese Medicine, Tianjin, China; 5 Acupuncture and Moxibustion Department, First Teaching Hospital of Tianjin University of Traditional Chinese Medicine, Tianjin, China; 6 Reproductive Center Department, First Teaching Hospital of Tianjin University of Traditional Chinese Medicine, Tianjin, China; King Abdulaziz University, SAUDI ARABIA

## Abstract

**Introduction:**

Oxidative stress is involved in the occurrence and development of multiple diseases. Acupuncture shows an excellent clinical efficacy in practical application but its mechanism remains unclear. This systematic review and meta-analysis was aimed at assessing the effect of acupuncture on oxidative stress in animal models.

**Methods:**

PubMed, Embase, and Web of Science database were retrieved for randomized controlled trials about acupuncture on oxidative stress in animal models from inception to August 2021. Two reviewers independently screened and extracted articles according to inclusion and exclusion criteria. We used the mean difference (MD)/standardized mean difference (SMD) to perform an effect size analysis and selected fixed-effect or random-effect models to pool the data, depending on a 95% confidence interval (CI).

**Results:**

A total of 12 studies comprising 125 samples were included in the quantitative meta-analysis. Compared with sham acupuncture, acupuncture (manual acupuncture, electropuncture, and laser acupuncture) reduced the level of malondialdehyde (SMD, −3.03; CI, −4.40, −1.65; p < 0.00001) and increased the levels of superoxide dismutase (SMD, 3.39; CI, 1.99, 4.79; p < 0.00001), glutathione peroxidase (SMD, 2.21; CI, 1.10, 3.32; p < 0.00001), and catalase (SMD, 2.80; CI, 0.57, 5.03; p = 0.01).

**Conclusion:**

This meta-analysis indicated that acupuncture can regulate oxidative stress by lowering the lipid peroxidation and activating the antioxidant enzyme system. In consideration of heterogeneity between studies, future studies should be performed by complying with strict standards and increasing sample size in animal experiments to reduce bias.

## Introduction

Oxidative stress is a classic biological process representing an imbalance between oxidative damage and oxidation resistance in vivo. Under normal physiological conditions, the generation of reactive oxygen species (ROS) in cells can not damage the biological function of the cells due to antioxidants released through cellular defense systems as a protective effect in vivo. A higher level of oxidative stress induced by various potential risk factors results in severe damage to all types of biomolecules when the levels of endogenous antioxidants are insufficient to quench the free radicals [1]. As a result, damage caused by oxidative stress can lead to the degeneration of proteins, lipids, and DNA/RNA, which in turn causes a series of pathological processes, including alteration of the genetic structure and DNA methylation, inhibition of cell proliferation and growth, the acceleration of cellular aging, and, ultimately cell death [2–4]. The modifications mentioned in the structure and function of cells can contribute, at least partially, to a variety of diseases including Alzheimer’s disease (AD), cardiovascular disease (CD), ischemic stroke (IS), diabetes mellitus (DM), spinal cord injury(SCI), and male infertility (MI) [5–9]. For instance, on account of the characteristics of vulnerability to oxidative damage and a deficiency of antioxidants, the cells in the brain can be easily attacked by ROS [10–12], which then induce the oxidation of lipids, proteins, and DNA/RNA-also a common pathological feature in AD [13]-finally accelerating neuronal degeneration [14]. Endothelial dysfunction has been confirmed to play a vital role in the occurrence and development of CD [15]. The release of ROS mediated by nicotinamide adenine dinucleotide phosphate (NADPH) oxidases can impact the availability of a critical endothelium-derived relaxing factor [16], nitric oxide (NO) [17], which in turn leads to the repair dysfunction of vascular endothelial cells [15]. As an essential pathological factor, oxidative stress also accelerates neuronal cell death and apoptosis [18, 19] after sudden interruption or severe reduction of the blood flow and oxygen supply to the brain, causing local edemas and elevated intracranial pressure. This phenomenon further hinders the perfusion of the brain tissues and results in an IS [20–22]. Several studies suggest that oxidative stress plays a key role in triggering insulin resistance and the subsequent disruption of insulin signaling [23–25], and oxidative stress can also influence the development of secondary diabetic complications involving neuropathy, nephropathy, vascular disease, and retinopathy [25, 26]. Oxidative stress can also deteriorate SCI. Free radical can be produced and released after SCI, which causes cell death and tissue damage and subsequently aggravating SCI [27]. Moreover, an excessive production of ROS in sperm can impact the ability of mitochondria to acquire energy, causing sperm membrane and DNA damage and thereby leading to a reduction in the potential of the sperm to fertilize an egg and generate a healthy embryo [28–31]. In summary, oxidative stress can be as the cause of the pathology among multiple diseases and the contributor to disease progression [32]. It is generally clear that oxidative stress is involved in pathological development and could be the underlying etiology of multiple diseases. Hence, the key to improve or cure diseases may depend on the supplementation of antioxidants or the regulation of the balance in oxidative stress through other methods based on this specific mechanism.

Acupuncture, with a long history of being practiced for over 3000 years in China, has shown a clinical efficacy in treating several diseases worldwide [33]. In particular, acupuncture generated an excellent efficacy in treatment of the diseases outlined above [34–38]. Over the past few decades, a majority of studies regarding the therapeutic mechanisms of acupuncture in vivo have focused on neuroregulation, immunoregulation, metabolism, and gastrointestinal system [39–42]. An increasing number of studies have suggested that acupuncture generates a positive effect in regulation of the oxidative stress status in animal models [43–47]. To the best of our knowledge, no systematic meta-analysis has been published to analyze the effect and mechanism of acupuncture on oxidative stress in animal experiments to date. Therefore, we performed a systematic review and meta-analysis to investigate the experimental data that support the oxidation resistance of acupuncture with a particular focus on the related indicators.

## Materials and methods

This study was conducted by following the guidelines of the Preferred Reporting Items for Systematic Reviews and Meta-analyses (PRISMA) [48] and was registered in the PROSPERO database (registration number: CRD42021256081).

### Search strategy

Comprehensive article searching was undertaken by two authors independently in the PubMed, Embase, and Web of Science databases from inception to August 2021 with no limitation on publication language. To identify any additional relevant articles, the two observers manually reviewed the lists of references in the selected articles. There were no limits on the publication data.

The whole search strategies (mesh terms and all fields) in PubMed were as follows: (Animal Model OR Animal Models OR Laboratory Animal Models OR Laboratory Animal Model OR Experimental Animal Models OR Animal OR Animal Models, Experimental OR Experimental Animal Model) AND (Acupuncture) AND (Oxidative Stresses OR Antioxidative Stress OR Antioxidative Stresses OR Anti-oxidative Stress OR Anti oxidative Stress OR Anti-oxidative Stresses OR Oxidative Damage OR Oxidative Damages OR Oxidative Stress Injury OR Oxidative Stress Injuries OR Oxidative Injury OR Oxidative Injuries OR Oxidative Cleavage OR Oxidative Cleavages OR Oxidative DNA Damage OR Oxidative DNA Damages OR DNA Oxidative Damage OR DNA Oxidative Damages OR Oxidative and Nitrative Stress OR Oxidative Nitrative Stress OR Oxidative Nitrative Stresses OR Nitro-Oxidative Stress OR Nitro Oxidative Stress OR Nitro-Oxidative Stresses) AND (Sham acupuncture).

In Embase, the search string was (animal model:ab,ti OR animal disease model:ab,ti OR animal models:ab,ti) AND (acupuncture:ab,ti OR acupuncture therapy:ab,ti OR shonishin:ab,ti) AND (oxidative stress:ab,ti OR oxidant stress:ab,ti OR oxidant stresses:ab,ti OR oxidative stresses:ab,ti) AND (sham acupuncture:ab,ti).

### Inclusion and exclusion criteria

The inclusion criteria applied to the study selection were as follows: (1) animal models with diseases caused by oxidative stress; (2) any type of acupuncture treatment with explicit instructions for acupoint selection, intensity, duration of treatment, and period (manual acupuncture: steel needles inserted into specific acupoints based on the meridian and collateral theory in the form of intermittent rotation; electropuncture: implemented combined with electrical stimulation on needles, in particular the strength of the electric current or voltage; laser acupuncture: operated by focusing irradiation at specific points with a low intensity laser); (3) comparisons with a control group that received a sham acupuncture intervention; and (4) any species, sex, weight, or age.

The exclusion criteria were as follows: (1) animal experiments in vitro or ex vivo, and studies in humans or silicon models; (2) combinations with other interventions (traditional Chinese medicine decoction, moxibustion, Chinese patent medicine, etc.); (3) case reports, literature reviews, and conference abstracts; and (4) full texts of studies not available.

### Study selection

After removing the duplicates, two reviewers screened the titles and abstracts to select the related studies to be imported into EndNote X7. Full text screening was then applied to identify the unique articles meeting the inclusion criteria. If there was a disagreement between the reviewers, it was resolved by consulting a third researcher through rigorous discussions.

### Data extraction

Information regarding each included study (e.g., authors, publication year, species, weight, acupoint selection, intervention, frequency or intensity, outcome measures, and treatment duration) was extracted by two reviewers independently. If the data were presented in the form of a graph, GetData Graph Digitizer (http://getdata-graph-digitizer.com/)(2021.9.30) was used to extract the numerical data from the diagrams [49].

### Risk of bias assessment

The SYRCLE RoB tool [50] was used independently by the two reviewers to evaluate the risk of bias (RoB). The tool contains 10 items involved in six aspects of bias (selection bias, performance bias, detection bias, attrition bias, reporting bias, and other biases). Scores of ‘yes’, ‘no’, and ‘unsure’ separately indicate a ‘low’, ‘high’, and ‘unclear’ RoB, respectively, and were shown on the Cochrane RoB tool [51].

### Data analysis

The experimental group (manual acupuncture, electropuncture, and laser acupuncture) and control group (sham acupuncture) data from the included studies were extracted and imported into Revman 5.3 software. When the outcome measures of all the included studies were on the same scale, the mean difference (MD) was used to perform the effect size analysis. Otherwise, the standardized mean difference (SMD) was used. Confidence intervals (CIs) of 95% were calculated for the effects of acupuncture on oxidative stress. The heterogeneity among the studies was classified according to the I^2^ test. When the I^2^ was ≤50% (low heterogeneity), a fixed-effect model was used. When the I^2^ was >50% (high heterogeneity), a random-effect model was used. When the subgroups comprised at least two independent comparisons, subgroup analyses were performed. A sensitivity analysis was conducted to account for the risk of bias through a leave-one-out method operated in OpenMeta (Analyst) software, represented by a leave-one-out forest plot.

### Publication bias

We implemented the assessment of publication bias using a visual inspection of the funnel plot asymmetry and Egger’s test of asymmetry [52]. If there were fewer than 10 studies associated with one outcome, the power of the assessment was too low to be performed according to the Cochrane recommendations. Egger’s test of asymmetry was also invalid on the condition that the number of included studies was fewer than 20.

## Results

### Study selection

**Fig 1:**
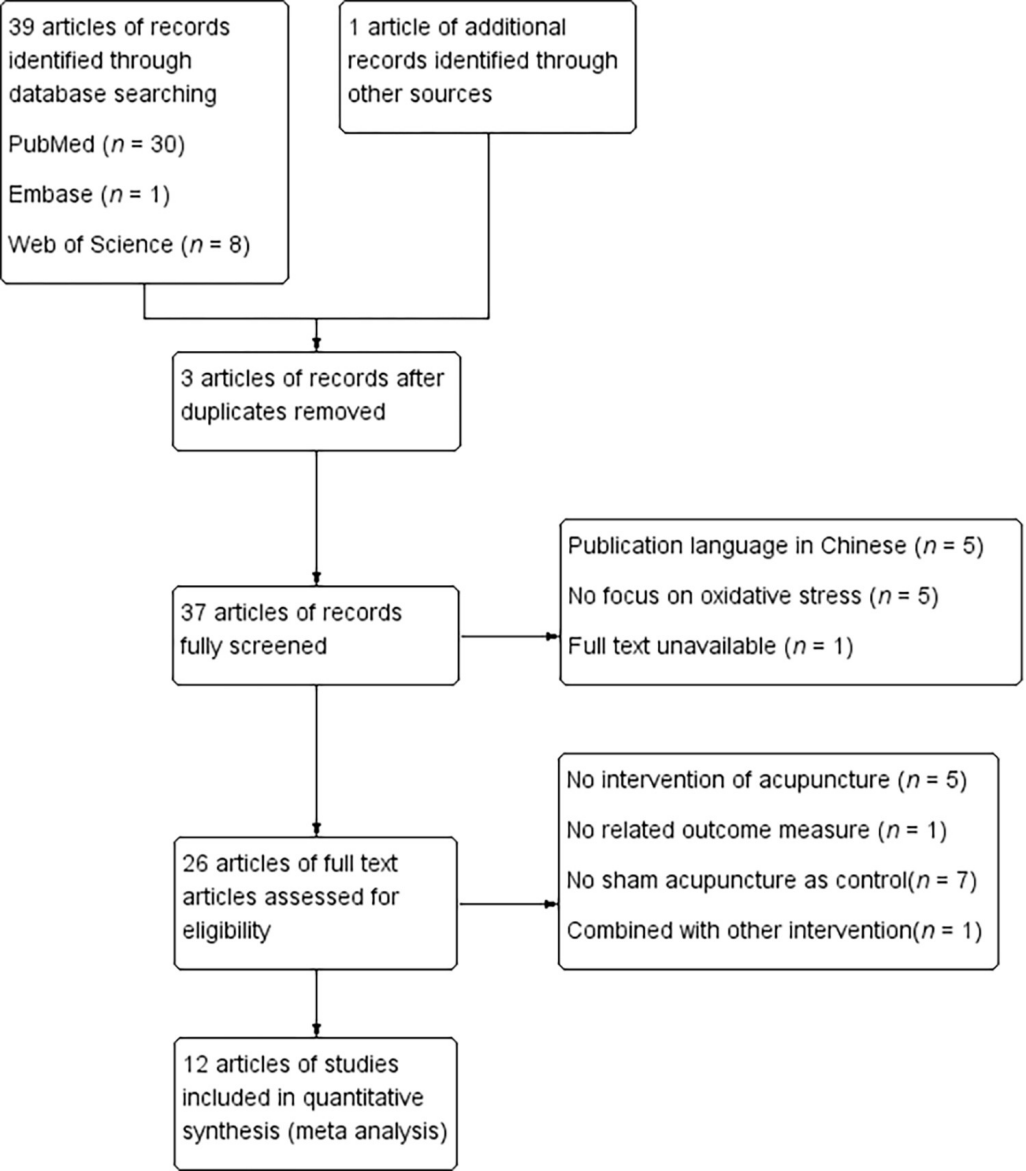
Flow diagram of the systematic review and article search results.

After a comprehensive search for articles in the databases, 40 articles were initially identified according to their titles and abstracts. Following a full text screening, several were eliminated based on the following reasons: duplicate publication (n = 3), publication language in Chinese (n = 5), not providing an intervention of any type of acupuncture (n = 5), not focusing on oxidative stress (n = 5), no related outcome measure provided (n = 1), no sham acupuncture as the control (n = 7), combined with another intervention (oral drugs) (n = 1), and full text unavailable (n = 1). Ultimately, a total of 12 studies comprising 125 animal models were included in the quantitative meta-analysis. The flow of searching the databases is displayed in Fig 1.

### Study characteristics

The characteristics of the 12 included studies are listed in Table 1. Of these studies, the animal models were all rats or mice, but of different breeds and ages. The number of samples per group ranged from 6 to 20. Five studies experimented with electroacupuncture (EA) [53–57], four with manual acupuncture (MA) [58–61], and three with laser acupuncture (LA) [62–64]. Five studies sampled the hippocampal tissues from the rats for detection [55, 58, 59, 64], two used plasma [56, 57], two used a homogenate of the brain tissues [54, 61], one used the spinal cord [53], one used the prefrontal cortex [61], and one used the cerebral cortex [63].

**Table 1:** Characteristics of the included studies.

Author	Species	Weight	Num	Exp	Con	Sample	Acupoint	Frequency/	Indicators	Duration (Day)
(Year)	(Sex)	(g)					Selection	Intensity		
Alvarado-Sanchez et al. [53] (2019)	Long Evans rats (female)	250–300	14	EA	SA	Spinal cord	GV4	2Hz/100 Hz	MDA	?
									H2O2	
								5.2 mA		
									TBARS	
Siu et al. [54] (2005)	Sprague–Dawley rats (male)	330–350	6	EA	SA	Homogenate of brain tissue	GB20	2 Hz	TR	14
									Trx	
							ST36	0.7 V		
									NADPH	
Li et al. [55] (2020)	Sprague–Dawley rats (male)	250–300	20	EA	SA	Hippocampal tissues	LI11,	2Hz/15Hz	MDA	10
							ST36			
							DU20	1.5 mA	SOD	
Leung et al. [56] (2016)	SHRs (male)	?	8	EA	SA	Plasma	ST36	2 Hz	NADPH	30
							LR3	2 mA		
Tian et al. [57] (2018)	C57BL/6 wild-type mice (male)	?	12	EA	SA	Plasma	ST36	10 Hz	MDA	32
								1–3 mA		
Chang et al. [58] (2019)	Senescence-resistant mouse strain 8 (male)	?	10	MA	SA	Hippocampal tissues	CV17	?	SOD	14
							CV12			
							CV6			
									GSH-Px	
							SP10			
							ST36			
Liu et al. [59] (2006)	Wistar rats (male)	340 ± 40	9	MA	SA	Hippocampal tissues	CV17	Twisted at the speed of twice a second for 30 s	SOD	14
							CV12			
							CV6		CAT	
							ST36		GSH-Px	
							SP10			
Phunchago et al. [60] (2014)	Wistar rats (male)	180–220	6	MA	SA	Homogenate of brain tissue	HT7	Twisted at the speed of twice a second for 60 s	MDA	14
									SOD	
									GSH-Px	
									CAT	
Fei-yi Z et al. [61] (2021)	Sprague–Dawley rats (male)	200 ± 20	14	MA	SA	Prefrontal cortex	GV20	?	MDA	18
							HT7			
							SP6		SOD	
							GV29		GSH-Px	
Sutalangka et al. [62] (2013)	Wistar rats (male)	180–220	6	LA	SA	Hippocampal tissues	HT7	405 nm	MDA	14
									SOD	
								100 mW	GSH-Px	
									CAT	
Jittiwat [63] (2017)	Wistar rats (male)	300–350	10	LA	SA	Cerebral cortex	GV20	810 nm	MDA	14
									SOD	
								100 mW	GSH-Px	
									CAT	
Jittiwat [64] (2019)	Wistar rats (male)	300–350	10	LA	SA	Hippocampal tissues	GV20	810 nm	SOD	14
								100 μm	GSH-Px	

?: not mentioned; SHRs: spontaneously hypertensive rats; EA: electroacupuncture; LA: laser acupuncture; MA: manual acupuncture; SA: sham acupuncture; MDA: malondialdehyde; TBARS: thiobarbituric acid reaction substance; GSH-Px: glutathione peroxidase; SOD: superoxide dismutase; GSH: glutathione; CAT: catalase; TR: thioredoxin reductase; Trx: thioredoxin; NADPH: nicotinamide adenine dinucleotide phosphate.

### Risk-of-bias assessment

**Fig 2:**
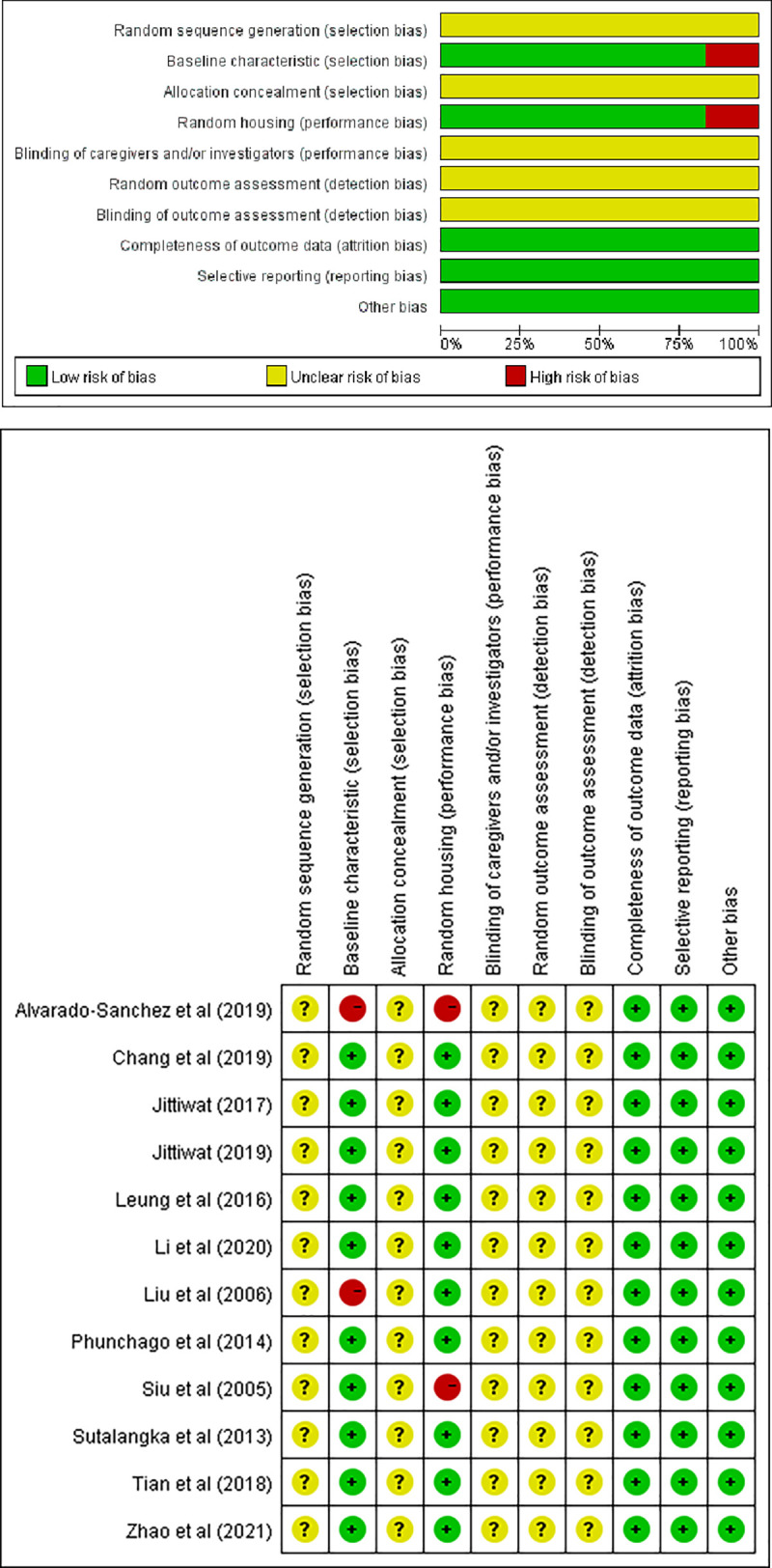
Risk of bias. (a) Following the SYRCLE tool, each risk-of-bias item is displayed as a percentage according to all included studies. (b) Individual risk of bias of the 10 items in the SYRCLE tool on all included studies, representing ‘yes’, ‘no’, or ‘unclear’.

The risk-of-bias assessment of the included studies is shown in Fig 2a and the individual scores for the 10 items of each study are presented in Fig 2b. In total, all 12 studies described a random allocation but did not provide specific random methods, which resulted in all being classified “unclear”. Only two studies [53, 59] did not describe the feeding conditions to ensure comparability of the baseline characteristics between the two groups. The risk of random housing was high in two studies [53, 54]. Across the studies, insufficient information led to an uncertainty of the risk of bias regarding the blinding of caregivers as well as the randomness and blinding of the outcome assessment. All studies recorded a complete outcome simultaneously without bias from other sources.

### Data extraction

Only two studies [58, 61] provided detailed data that were represented numerically. Other studies presented the experimental data graphically; therefore, GetData Graph Digitizer was used to obtain the numerical data.

### Malondialdehyde (MDA)

**Fig 3:**
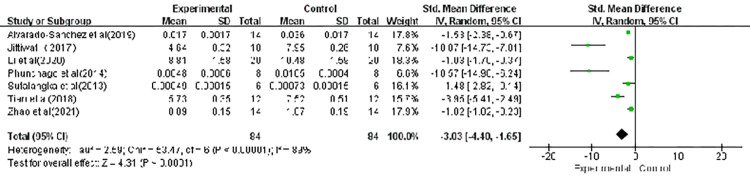
Forest plot showing the effect of acupuncture on MDA levels. CI: confidence interval; IV: inverse variance; MD: mean difference; SD: standard deviation; WMD: weighted mean difference.

Seven of the 12 included studies measured the malondialdehyde (MDA) level and these data are shown in Fig 3. Given the high heterogeneity among the included studies, a random-effect model was used to pool the data. Compared with sham acupuncture, acupuncture significantly decreased the level of MDA (SMD, -3.03; CI, -4.40, -1.65; *p* < 0.00001). A sensitivity analysis was performed and illustrated that the effect sizes were stable; the elimination of a single study did not impact the significance.

### Superoxide Dismutase (SOD)

**Fig 4:**
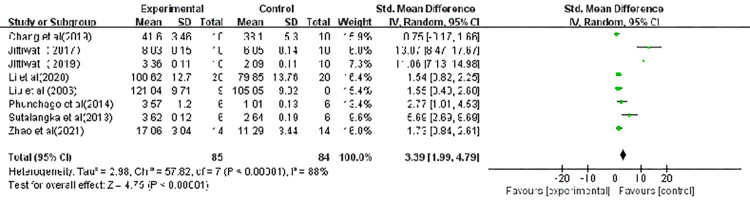
Forest plot showing the effect of acupuncture on SOD levels. Note: CI: confidence interval; IV: inverse variance; MD: mean difference; SD: standard deviation; WMD: weighted mean difference.

Eight of the included studies measured the superoxide dismutase (SOD) level and the pooled data of these are presented in Fig 4. Similarly, a random-effect model was performed due to a high level of heterogeneity between the individual studies. In the meta-analysis, acupuncture was associated with a significant improvement on the SOD level (SMD, 3.39; CI, 1.99, 4.79; p < 0.00001). A sensitivity analysis was performed and illustrated that the effect sizes were stable; the elimination of a single study did not impact the significance.

### Glutathione Peroxidase (GSH-Px)

**Fig 5:**
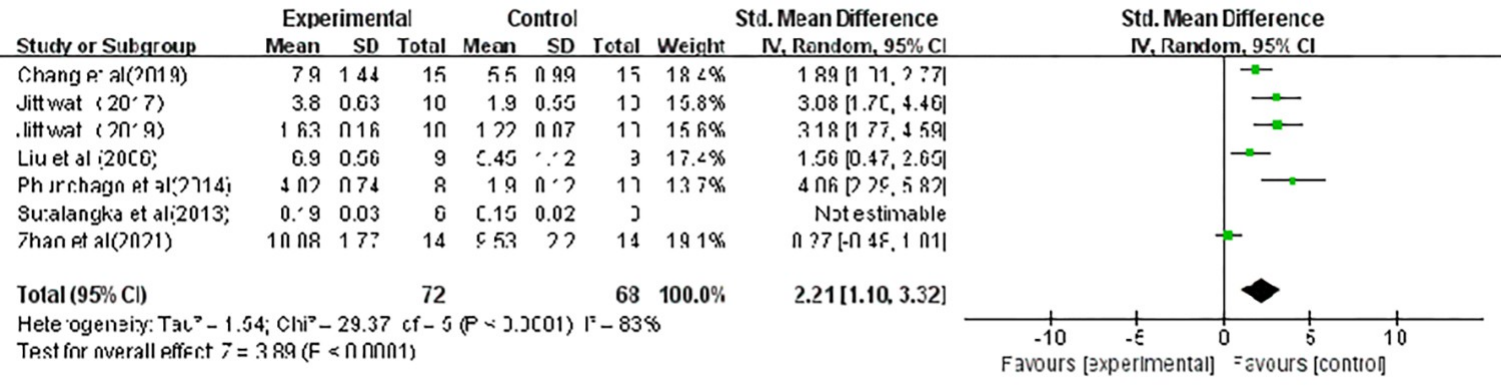
Forest plot showing the effect of acupuncture on GSH-Px levels. Note: CI: confidence interval; IV: inverse variance; MD: mean difference; SD: standard deviation; WMD: weighted mean difference.

Seven of the included studies measured the glutathione peroxidase (GSH-Px) level and the pooled data of these seven studies are displayed in Fig 5. Acupuncture increased the level of GSH-Px (SMD, 2.21; CI, 1.10, 3.32; p < 0.00001). A sensitivity analysis was performed and illustrated that the effect sizes were stable; the elimination of a single study did not impact the significance.

### Catalase (CAT)

**Fig 6:**
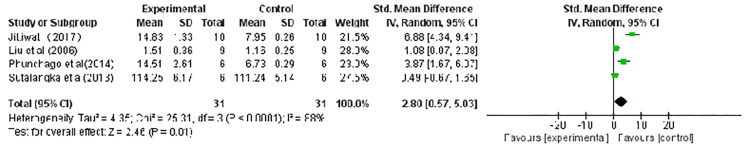
Forest plot showing the effect of acupuncture on CAT levels. Note: CI: confidence interval; IV: inverse variance; MD: mean difference; SD: standard deviation; WMD: weighted mean difference.

Four of the included studies measured the catalase (CAT) level and the pooled data of these are displayed in Fig 6. Acupuncture increased the level of CAT (SMD, 2.80; CI, 0.57, 5.03; p = 0.01). A sensitivity analysis was performed and illustrated that the effect sizes was stable; the elimination of a single study did not impact the significance.

### Subgroup analysis

Subgroup analyses were performed based on the type of intervention and the species used in the experimental animals. Four studies [58–61] that used manual acupuncture, two studies [55, 61] that used Sprague–Dawley rats and evaluated MDA, and three studies [62–64] that used laser acupuncture and evaluated GSH-Px showed a low heterogeneity between each other. The pooled data of the subgroup analyses are shown in Table 2.

**Table 2:** Subgroup analyses of studies using different types of acupuncture and species of experimental animals.

Indicator	Intervention/Species	SMD (95% CI)	I2	p (Heterogeneity)
MDA	Electropuncture	−2.02 (−3.38, −0.65)	84%	0.002
	Laser acupuncture	−5.99 (−15.20, 3.21)	95%	< 0.00001
	Wistar rats	−7.44 (−14.71, −0.18)	94%	< 0.00001
SOD	Manual acupuncture	1.51 (0.82, 2.21)	38%	0.19
	Laser acupuncture	9.70 (5.12, 14.28)	77%	0.01
	Wistar rats	6.30 (2.81, 9.78)	91%	< 0.00001
	Sprague–Dawley rats	1.61 (1.06, 2.17)	0	0.75
GSH-Px	Manual acupuncture	1.78 (0.48, 3.07)	84%	0.0003
	Laser acupuncture	2.55 (1.44, 3.67)	49%	0.14
	Wistar rats	2.56 (1.60, 3.51)	59%	0.05
CAT	Manual acupuncture	2.29 (−0.42, 5.01)	80%	0.02
	Laser acupuncture	3.58 (−2.68, 9.84)	95%	< 0.00001

Note: MDA: malondialdehyde; SOD: superoxide dismutase; GSH-Px: glutathione peroxidase; CAT: catalase.

## Discussion

In this study, we demonstrated that acupuncture can regulate oxidative stress in animal models in different organs including the brain, vessels, stomach, and spinal nerves compared with sham acupuncture. Twelve studies were eligible for the meta-analysis, which showed that acupuncture could significantly reduce the MDA level and increase the SOD, GSH-Px, and CAT levels. A high level of lipid peroxidation can overwhelm antioxidant defent system in vivo and induce cell apoptosis or other pathological reaction [65]. This process will elevate the concentration of MDA, which can reflect the increase of free radical production and the degree of oxidative stress [66]; SOD, GSH-Px and CAT are all excellent antioxidants in vivo and participation of antioxidant systems and play a part through eliminating oxygen free radicals [67]. Hence, we confirmed that acupuncture stimulating specific acupoints decreased the levels of lipid peroxidation and activated the inherent antioxidant enzyme system to balance the oxidative stress status in several tissues and organs. A data analysis of the relevant indicators demonstrated that acupuncture plays an important role in regulating the oxidative stress reaction; furthermore, it provides an excellent curative effect through multiple target points.

From a clinical point of view, independent of the type of intervention applied (MA, EA, or LA), all studies provided stimulation at a specific point and produced an effect on the tissue. We pooled the data from the included studies using different types of intervention. Based on the subgroup analysis, we observed that the results of LA on MDA (SMD, -5.99; CI, -15.20, 3.21; p = 0.20), and MA (SMD, 2.29; CI, -0.42, 5.01; p = 0.10) and LA (SMD, 3.58; CI, -2.68, 9.84; p = 0.26) on CAT had no statistical differences. Apart from the subgroup analysis on SOD, all subgroups overlapped on the confidence interval, which suggest that there were interactions between the variables. Therefore, the results of the subgroup analysis did not affect the results of the comprehensive analysis.

Despite applying an experimental design and being highly controlled, there was still considerable heterogeneity among the studies included in this article. The subgroup analysis based on the different types of intervention and animal model indicated that heterogeneity still existed between the studies. This phenomenon may be due to differences in the forms of animal rearing, experiment reagents, sampling, and acupuncture prescriptions. As a characteristic of the acupoint selection in acupuncture, a systematic and standardized treatment protocol may not have been implemented. Therefore, a greater uniformity of the protocol design and of the animal models would have minimized the heterogeneity between the studies, enhanced the comparability of results, reduced experimental errors, and increased the grade of evidence. However, because of the small sample size, this subgroup analysis lacked statistical power.

Over the years, the therapeutic mechanism of acupuncture has remained unclear and, as a result, has always been a research focus [68]. The reason for this phenomenon might be that the practical application of this ancient technology is based on the meridian and collateral theory, which is beyond understanding and unobservable in the human anatomy [69]. Considering its beneficial effects, it is necessary to explore and establish the potential mechanisms of acupuncture in treating diseases. With cumulative animal studies being performed [43, 70–72], the relationship between acupuncture and oxidative stress has become clear. Despite a high heterogeneity between the included studies, the trend of improvement of oxidative stress by acupuncture was displayed through the pooled data, which were consistent with previous studies [73, 74]. Resisting oxidative stress is known to involve several aspects [75], such as (i) the inhibition of the production of ROS; (ii) the elimination of ROS by antioxidant enzymes or another signal pathway; and (iii) the repairing of proteins, lipids, or DNA attacked by ROS. A study indicated that EA stimulation at GV20 in diabetic rats with a cerebral ischemia could inhibit the activation of NOX, a major ROS-producing enzyme, and lower the MDA content and ROS formation [76]. Another study reported that manual acupuncture at Tanzhong (CV17), Zhongwan (CV12), Qihai (CV6), Sanyinjiao (ST36), and Xuehai (SP10) promoted the activities of the total SOD and decreased the level of MDA in mitochondria [77]. EA stimulation at GV20 and ST36 attenuated oxidative stress via increased CAT and SOD activity in the serum and hippocampus [78], which suggested that acupuncture could regulate oxidative stress through antioxidant enzymes in vivo. Meanwhile, EA stimulation at ST36 and SP6 can lower the total SOD activity and inhibit the H_2_O_2_ and MDA level in corpus striatum[79]. At a molecular level, acupuncture similarly has been found to repair proteins, lipids, or DNA attacked by ROS [44].

As discussed, exogenous supplementation of antioxidants plays an essential role in clinical practice. The application of various antioxidants has been proven with excellent clinical effects [80, 81]. Compared with exogenous antioxidants that need to be administered orally, acupuncture has several advantages such as it not being metabolized in vivo as well as economy and acceptability. Although this meta-analysis focused on animal studies, the data from this study support the function of acupuncture on oxidative stress and point to a direction for future clinical studies as a basis or guidance for human disease.

To our knowledge, this is the first systematic review of the effects of acupuncture on oxidative stress in animal studies. A comprehensive search was applied in multiple databases for the full texts of all identified articles. The SYRCLE RoB tool was used to assess the quality of the studies and data related to oxidative stress were extracted. Subgroup and sensitivity analyses were performed to validate our discoveries.

There are a few limitations to this study. First, the published language was limited to English. Second, one study was excluded as the full text was unavailable. Finally, the quality assessment using the SYRCE RoB tool reflected that the included studies did not provide sufficient information to reduce the risk of performance and detection bias.

## Conclusion

In conclusion, we observed that acupuncture significantly decreases the level of MDA and increases the levels of SOD, GSH-Px, and CAT. The data from existing experimental studies suggest that acupuncture can regulate oxidative stress status among multiple organs and tissues in animal models. However, more studies, especially clinical studies, are still needed to further explore and justify the oxidation resistance of acupuncture. All animal studies had major methodological limitations including a small sample size, performance bias, and detection bias. Therefore, future studies should be performed according to strict standards to reduce bias and by increasing the sample size in animal experiments.

## Supporting information

S1 File PRISMA_2020_checklist. (DOCX)

S1 Fig (SOD) Leave-one-out_Forest_Plot. (TIF)

S2 Fig (MDA) Leave-one-out_Forest_Plot. (TIF)

S3 Fig (CAT) Leave-one-out_Forest_Plot. (TIF)

S4 Fig (GSH-Px) Leave-one-out_Forest_Plot. (TIF)

## References

[1] Willcox JK, Ash SL, Catignani GL. Antioxidants and prevention of chronic disease. Crit Rev Food Sci Nutr. 2004; 44(4):275–95. doi: 10.1080/10408690490468489 15462130

[2] Kreuz S, Fischle W. Oxidative stress signaling to chromatin in health and disease. Epigenomics. 2016;8(6):843–62. doi: 10.2217/epi-2016-0002 27319358 PMC5619053

[3] Burdon RH. Superoxide and hydrogen peroxide in relation to mammalian cell proliferation. Free Radic Biol Med. 1995;18(4):775–94. doi: 10.1016/0891-5849(94)00198-s 7750801

[4] Hensley K, Robinson KA, Gabbita SP, Salsman S, Floyd RA. Reactive oxygen species, cell signaling, and cell injury. Free Radic Biol Med. 2000;28(10):1456–62. doi: 10.1016/s0891-5849(00)00252-5 10927169

[5] Tamagno E, Guglielmotto M, Vasciaveo V, Tabaton M. Oxidative Stress and Beta Amyloid in Alzheimer’s Disease. Which Comes First: The Chicken or the Egg? Antioxidants (Basel). 2021;10(9):1479. doi: 10.3390/antiox10091479 34573112 PMC8468973

[6] Juul F, Vaidean G, Parekh N. Ultra-processed Foods and Cardiovascular Diseases: Potential Mechanisms of Action. Adv Nutr. 2021;12(5):1673–1680. doi: 10.1093/advances/nmab049 33942057 PMC8483964

[7] Orellana-Urzúa S, Rojas I, Líbano L, Rodrigo R. Pathophysiology of Ischemic Stroke: Role of Oxidative Stress. Curr Pharm Des. 2020;26(34):4246–4260. doi: 10.2174/1381612826666200708133912 32640953

[8] Zhang P, Li T, Wu X, Nice EC, Huang C, Zhang Y. Oxidative stress and diabetes: antioxidative strategies. Front Med. 2020;14(5):583–600. doi: 10.1007/s11684-019-0729-1 32248333

[9] Agarwal A, Parekh N, Panner Selvam MK, Henkel R, Shah R, Homa ST, et al. Male Oxidative Stress Infertility (MOSI): Proposed Terminology and Clinical Practice Guidelines for Management of Idiopathic Male Infertility. World J Mens Health. 2019;37(3):296–312. doi: 10.5534/wjmh.190055 31081299 PMC6704307

[10] Butterfield DA, Castegna A, Lauderback CM, Drake J. Evidence that amyloid beta-peptide-induced lipid peroxidation and its sequelae in Alzheimer’s disease brain contribute to neuronal death. Neurobiol Aging. 2002;23(5):655–64. doi: 10.1016/s0197-4580(01)00340-2 12392766

[11] Butterfield DA, Reed T, Newman SF, Sultana R. Roles of amyloid beta-peptide-associated oxidative stress and brain protein modifications in the pathogenesis of Alzheimer’s disease and mild cognitive impairment. Free Radic Biol Med. 2007;43(5):658–77. doi: 10.1016/j.freeradbiomed.2007.05.037 17664130 PMC2031860

[12] Ahmad W, Ijaz B, Shabbiri K, Ahmed F, Rehman S. Oxidative toxicity in diabetes and Alzheimer’s disease: mechanisms behind ROS/ RNS generation. J Biomed Sci. 2017;24(1):76. doi: 10.1186/s12929-017-0379-z 28927401 PMC5606025

[13] Chen Z, Zhong C. Oxidative stress in Alzheimer’s disease. Neurosci Bull. 2014;30(2):271–81. doi: 10.1007/s12264-013-1423-y 24664866 PMC5562667

[14] Wang X, Wang W, Li L, Perry G, Lee HG, Zhu X. Oxidative stress and mitochondrial dysfunction in Alzheimer’s disease. Biochim Biophys Acta. 2014;1842(8):1240–7. doi: 10.1016/j.bbadis.2013.10.015 24189435 PMC4007397

[15] Senoner T, Dichtl W. Oxidative Stress in Cardiovascular Diseases: Still a Therapeutic Target? Nutrients. 2019;11(9):2090. doi: 10.3390/nu11092090 31487802 PMC6769522

[16] Förstermann U. Nitric oxide and oxidative stress in vascular disease. Pflugers Arch. 2010;459(6):923–39. doi: 10.1007/s00424-010-0808-2 20306272

[17] Brandes RP, Weissmann N, Schröder K. NADPH oxidases in cardiovascular disease. Free Radic Biol Med. 2010;49(5):687–706. doi: 10.1016/j.freeradbiomed.2010.04.030 20444433

[18] Crack PJ, Taylor JM. Reactive oxygen species and the modulation of stroke. Free Radic Biol Med. 2005;38(11):1433–44. doi: 10.1016/j.freeradbiomed.2005.01.019 15890617

[19] Gürsoy-Ozdemir Y, Can A, Dalkara T. Reperfusion-induced oxidative/nitrative injury to neurovascular unit after focal cerebral ischemia. Stroke. 2004;35(6):1449–53. doi: 10.1161/01.STR.0000126044.83777.f4 15073398

[20] Allen CL, Bayraktutan U. Oxidative stress and its role in the pathogenesis of ischaemic stroke. Int J Stroke. 2009;4(6):461–70. doi: 10.1111/j.1747-4949.2009.00387.x 19930058

[21] Bektas H, Wu TC, Kasam M, Harun N, Sitton CW, Grotta JC, et al. Increased blood-brain barrier permeability on perfusion CT might predict malignant middle cerebral artery infarction. Stroke. 2010;41(11):2539–44. doi: 10.1161/STROKEAHA.110.591362 20847316 PMC3412880

[22] Kahles T, Kohnen A, Heumueller S, Rappert A, Bechmann I, Liebner S, et al. NADPH oxidase Nox1 contributes to ischemic injury in experimental stroke in mice. Neurobiol Dis. 2010;40(1):185–92. doi: 10.1016/j.nbd.2010.05.023 20580928

[23] Matsuzawa-Nagata N, Takamura T, Ando H, Nakamura S, Kurita S, Misu H, et al. Increased oxidative stress precedes the onset of high-fat diet-induced insulin resistance and obesity. Metabolism. 2008;57(8):1071–7. doi: 10.1016/j.metabol.2008.03.010 18640384

[24] Houstis N, Rosen ED, Lander ES. Reactive oxygen species have a causal role in multiple forms of insulin resistance. Nature. 2006;440(7086):944–8. doi: 10.1038/nature04634 16612386

[25] Gerrits EG, Alkhalaf A, Landman GW, van Hateren KJ, Groenier KH, Struck J, et al. Serum peroxiredoxin 4: a marker of oxidative stress associated with mortality in type 2 diabetes (ZODIAC-28). PLoS One. 2014;9(2):e89719. doi: 10.1371/journal.pone.0089719 24586984 PMC3934910

[26] Phillips M, Cataneo RN, Cheema T, Greenberg J. Increased breath biomarkers of oxidative stress in diabetes mellitus. Clin Chim Acta. 2004;344(1–2):189–94. doi: 10.1016/j.cccn.2004.02.025 15149888

[27] Jiang K, Sun Y, Chen X. Mechanism Underlying Acupuncture Therapy in Spinal Cord Injury: A Narrative Overview of Preclinical Studies. Front Pharmacol. 2022;13:875103. doi: 10.3389/fphar.2022.875103 35462893 PMC9021644

[28] Aitken RJ. Reactive oxygen species as mediators of sperm capacitation and pathological damage. Mol Reprod Dev. 2017;84(10):1039–1052. doi: 10.1002/mrd.22871 28749007

[29] Agarwal A, Cho CL, Esteves SC, Majzoub A. Reactive oxygen species and sperm DNA fragmentation. Transl Androl Urol. 2017;6(Suppl 4):S695–S696. doi: 10.21037/tau.2017.05.40 29082952 PMC5643645

[30] Muratori M, Tamburrino L, Marchiani S, Cambi M, Olivito B, Azzari C, et al. Investigation on the Origin of Sperm DNA Fragmentation: Role of Apoptosis, Immaturity and Oxidative Stress. Mol Med. 2015;21(1):109–22. 25786204 10.2119/molmed.2014.00158 PMC4461587

[31] Truong T, Gardner DK. Antioxidants improve IVF outcome and subsequent embryo development in the mouse. Hum Reprod. 2017;32(12):2404–2413. doi: 10.1093/humrep/dex330 29136144

[32] Forman HJ, Zhang H. Targeting oxidative stress in disease: promise and limitations of antioxidant therapy. Nat Rev Drug Discov. 2021;20(9):689–709. doi: 10.1038/s41573-021-00233-1 34194012 PMC8243062

[33] Chon TY, Lee MC. Acupuncture. Mayo Clin Proc. 2013;88(10):1141–6. doi: 10.1016/j.mayocp.2013.06.009 24079683

[34] Jia Y, Zhang X, Yu J, Han J, Yu T, Shi J, et al. Acupuncture for patients with mild to moderate Alzheimer’s disease: a randomized controlled trial. BMC Complement Altern Med. 2017;17(1):556. doi: 10.1186/s12906-017-2064-x 29284465 PMC5747102

[35] Palma F, Fontanesi F, Neri I, Xholli A, Facchinetti F, Cagnacci A. Blood pressure and cardiovascular risk factors in women treated for climacteric symptoms with acupuncture, phytoestrogens, or hormones. Menopause. 2020;27(9):1060–1065. doi: 10.1097/GME.0000000000001626 32852460

[36] Li M, Zhang B, Meng Z, Sha T, Han Y, Zhao H, et al. Effect of Tiaoshen Kaiqiao acupuncture in the treatment of ischemic post-stroke depression: a randomized controlled trial. J Tradit Chin Med. 2017;37(2):171–8. doi: 10.1016/s0254-6272(17)30041-9 29960288

[37] Mooventhan A, Ningombam R, Nivethitha L. Effect of bilateral needling at an acupuncture point, ST-36 (Zusanli) on blood glucose levels in type 2 diabetes mellitus patients: A pilot randomized placebo controlled trial. J Complement Integr Med. 2020;17(3). doi: 10.1515/jcim-2019-0100 32406384

[38] Kucuk EV, Bindayi A, Boylu U, Onol FF, Gumus E. Randomised clinical trial of comparing effects of acupuncture and varicocelectomy on sperm parameters in infertile varicocele patients. Andrologia. 2016;48(10):1080–1085. doi: 10.1111/and.12541 26791438

[39] Chavez LM, Huang SS, MacDonald I, Lin JG, Lee YC, Chen YH. Mechanisms of Acupuncture Therapy in Ischemic Stroke Rehabilitation: A Literature Review of Basic Studies. Int J Mol Sci. 2017;18(11):2270. doi: 10.3390/ijms18112270 29143805 PMC5713240

[40] Kim SK, Bae H. Acupuncture and immune modulation. Auton Neurosci. 2010;157(1–2):38–41. doi: 10.1016/j.autneu.2010.03.010 20399151

[41] Qu F, Cui Y, Zeng J, Zhang M, Qiu S, Huang X, et al. Acupuncture induces adenosine in fibroblasts through energy metabolism and promotes proliferation by activating MAPK signaling pathway via adenosine_3_ receptor. J Cell Physiol. 2020;235(3):2441–2451. 31556103 10.1002/jcp.29148

[42] Liu J, Huang H, Xu X, Chen JD. Effects and possible mechanisms of acupuncture at ST36 on upper and lower abdominal symptoms induced by rectal distension in healthy volunteers. Am J Physiol Regul Integr Comp Physiol. 2012;303(2):R209–17. doi: 10.1152/ajpregu.00301.2010 22592556 PMC3404632

[43] Yang B, Huang H, He Q, Lu W, Zheng L, Cui L. Tert-Butylhydroquinone Prevents Oxidative Stress-Mediated Apoptosis and Extracellular Matrix Degradation in Rat Chondrocytes. Evid Based Complement Alternat Med. 2021;2021:1905995. doi: 10.1155/2021/1905995 34925524 PMC8674040

[44] Chen CH, Hsieh CL. Effect of Acupuncture on Oxidative Stress Induced by Cerebral Ischemia-Reperfusion Injury. Antioxidants (Basel). 2020;9(3):248. doi: 10.3390/antiox9030248 32204376 PMC7139408

[45] Cheng M, Wu X, Wang F, Tan B, Hu J. Electro-Acupuncture Inhibits p66Shc-Mediated Oxidative Stress to Facilitate Functional Recovery After Spinal Cord Injury. J Mol Neurosci. 2020;70(12):2031–2040. doi: 10.1007/s12031-020-01609-5 32488847

[46] Yu YP, Ju WP, Li ZG, Wang DZ, Wang YC, Xie AM. Acupuncture inhibits oxidative stress and rotational behavior in 6-hydroxydopamine lesioned rat. Brain Res. 2010;1336:58–65. doi: 10.1016/j.brainres.2010.04.020 20399757

[47] Frantz AL, Regner GG, Pflüger P, Coelho VR, da Silva LL, Viau CM, et al. Manual acupuncture improves parameters associated with oxidative stress and inflammation in PTZ-induced kindling in mice. Neurosci Lett. 2017;661:33–40. doi: 10.1016/j.neulet.2017.09.044 28947384

[48] Page MJ, McKenzie JE, Bossuyt PM, Boutron I, Hoffmann TC, Mulrow CD, et al. The PRISMA 2020 statement: an updated guideline for reporting systematic reviews. BMJ. 2021;372:n71. doi: 10.1136/bmj.n71 33782057 PMC8005924

[49] Digitizer G.G. Getdata-Graph-Digitizer. 2020. [(cited 19 February 2021)]. Available from: http://getdata-graph-digitizer.com/

[50] Hooijmans CR, Rovers MM, de Vries RB, Leenaars M, Ritskes-Hoitinga M, Langendam MW. SYRCLE’s risk of bias tool for animal studies. BMC Med Res Methodol. 2014;14:43. doi: 10.1186/1471-2288-14-43 24667063 PMC4230647

[51] Higgins JP, Altman DG, Gøtzsche PC, Jüni P, Moher D, Oxman AD, et al; Cochrane Bias Methods Group; Cochrane Statistical Methods Group. The Cochrane Collaboration’s tool for assessing risk of bias in randomised trials. BMJ. 2011;343:d5928. 22008217 10.1136/bmj.d5928 PMC3196245

[52] Egger M, Davey Smith G, Schneider M, Minder C. Bias in meta-analysis detected by a simple, graphical test. BMJ. 1997;315(7109):629–34. doi: 10.1136/bmj.315.7109.629 9310563 PMC2127453

[53] Alvarado-Sanchez BG, Salgado-Ceballos H, Torres-Castillo S, Rodriguez-Silverio J, Lopez-Hernandez ME, Quiroz-Gonzalez S, et al. Electroacupuncture and Curcumin Promote Oxidative Balance and Motor Function Recovery in Rats Following Traumatic Spinal Cord Injury. Neurochem Res. 2019;44(2):498–506. doi: 10.1007/s11064-018-02704-1 30603981

[54] Siu FK, Lo SC, Leung MC. Electro-acupuncture potentiates the disulphide-reducing activities of thioredoxin system by increasing thioredoxin expression in ischemia-reperfused rat brains. Life Sci. 2005;77(4):386–99. doi: 10.1016/j.lfs.2004.10.069 15894008

[55] Li C, Yu TY, Zhang Y, Wei LP, Dong SA, Shi J, et al. Electroacupuncture Improves Cognition in Rats With Sepsis-Associated Encephalopathy. J Surg Res. 2020;256:258–266. doi: 10.1016/j.jss.2020.06.056 32712439

[56] Leung SB, Zhang H, Lau CW, Lin ZX. Attenuation of blood pressure in spontaneously hypertensive rats by acupuncture was associated with reduction oxidative stress and improvement from endothelial dysfunction. Chin Med. 2016;11(1):38. doi: 10.1186/s13020-016-0110-0 27582785 PMC5006281

[57] Tian L, Song S, Zhu B, Liu S. Electroacupuncture at ST-36 Protects Interstitial Cells of Cajal via Sustaining Heme Oxygenase-1 Positive M2 Macrophages in the Stomach of Diabetic Mice. Oxid Med Cell Longev. 2018;2018:3987134. doi: 10.1155/2018/3987134 29854081 PMC5944261

[58] Chang S, Guo X, Li G, Zhang X, Li J, Jia Y, et al. Acupuncture promotes expression of Hsp84/86 and delays brain ageing in SAMP8 mice. Acupunct Med. 2019;37(6):340–347. doi: 10.1136/acupmed-2017-011577 31412703

[59] Liu CZ, Yu JC, Zhang XZ, Fu WW, Wang T, Han JX. Acupuncture prevents cognitive deficits and oxidative stress in cerebral multi-infarction rats. Neurosci Lett. 2006;393(1):45–50. doi: 10.1016/j.neulet.2005.09.049 16236447

[60] Phunchago N, Wattanathorn J, Chaisiwamongkol K, Muchimapura S, Thukham-Mee W. Acupuncture reduces memory impairment and oxidative stress and enhances cholinergic function in an animal model of alcoholism. J Acupunct Meridian Stud. 2015;8(1):23–9. doi: 10.1016/j.jams.2014.11.008 25660441

[61] Fei-yi Z., Sheng-nan G., Yan X., Hong X., Guo-Hua W., Hua-ling S., et al. Investigation of acupuncture in improving sleep, cognitive and emotion based on attenuation of oxidative stress in prefrontal cortex in sleep-deprived rats. J. Acupunct. Tuina. Sci. 2021;19:157–166.

[62] Sutalangka C, Wattanathorn J, Muchimapura S, Thukham-Mee W, Wannanon P, Tong-un T. Laser acupuncture improves memory impairment in an animal model of Alzheimer’s disease. J Acupunct Meridian Stud. 2013;6(5):247–51. doi: 10.1016/j.jams.2013.07.001 24139462

[63] Jittiwat J. Laser Acupuncture at GV20 Improves Brain Damage and Oxidative Stress in Animal Model of Focal Ischemic Stroke. J Acupunct Meridian Stud. 2017;10(5):324–330. doi: 10.1016/j.jams.2017.08.003 29078967

[64] Jittiwat J. Baihui Point Laser Acupuncture Ameliorates Cognitive Impairment, Motor Deficit, and Neuronal Loss Partly via Antioxidant and Anti-Inflammatory Effects in an Animal Model of Focal Ischemic Stroke. Evid Based Complement Alternat Med. 2019;2019:1204709. doi: 10.1155/2019/1204709 30915140 PMC6409074

[65] Ayala A, Muñoz MF, Argüelles S. Lipid peroxidation: production, metabolism, and signaling mechanisms of malondialdehyde and 4-hydroxy-2-nonenal. Oxid Med Cell Longev. 2014;2014:360438. doi: 10.1155/2014/360438 24999379 PMC4066722

[66] Cherian D, Peter T, Narayanan A, Madhavan SS, Achammada S, Vynat GP. Malondialdehyde as a marker of oxidative stress in periodontitis patients. J Pharm Bioallied Sci. 2019;11(6):297–300. doi: 10.4103/JPBS.JPBS_17_19 31198357 PMC6555357

[67] Sies H, Berndt C, Jones DP. Oxidative Stress. Annu Rev Biochem. 2017, 86: 715–748. doi: 10.1146/annurev-biochem-061516-045037 28441057

[68] Zhuang Y, Xing JJ, Li J, Zeng BY, Liang FR. History of acupuncture research. Int Rev Neurobiol. 2013;111:1–23. doi: 10.1016/B978-0-12-411545-3.00001-8 24215915

[69] Effect Takahashi T. and mechanism of acupuncture on gastrointestinal diseases. Int Rev Neurobiol. 2013;111:273–94. 24215928 10.1016/B978-0-12-411545-3.00014-6

[70] Khongrum J, Wattanathorn J. Laser Acupuncture Improves Behavioral Disorders and Brain Oxidative Stress Status in the Valproic Acid Rat Model of Autism. J Acupunct Meridian Stud. 2015;8(4):183–91. doi: 10.1016/j.jams.2015.06.008 26276454

[71] Lima LP, de Oliveira Albuquerque A, de Lima Silva JJ, Medeiros Fd, de Vasconcelos PR, Guimarães SB. Electroacupuncture attenuates oxidative stress in random skin flaps in rats. Aesthetic Plast Surg. 2012;36(5):1230–5. doi: 10.1007/s00266-012-9926-x 22678136

[72] Liu Z, Niu W, Yang X, Wang Y. Effects of combined acupuncture and eugenol on learning-memory ability and antioxidation system of hippocampus in Alzheimer disease rats via olfactory system stimulation. J Tradit Chin Med. 2013;33(3):399–402. doi: 10.1016/s0254-6272(13)60186-7 24024340

[73] Yu JB, Shi J, Gong LR, Dong SA, Xu Y, Zhang Y, et al. Role of Nrf2/ARE pathway in protective effect of electroacupuncture against endotoxic shock-induced acute lung injury in rabbits. PLoS One. 2014;9(8):e104924. doi: 10.1371/journal.pone.0104924 25115759 PMC4130631

[74] Zhang X, Wu B, Nie K, Jia Y, Yu J. Effects of acupuncture on declined cerebral blood flow, impaired mitochondrial respiratory function and oxidative stress in multi-infarct dementia rats. Neurochem Int. 2014;65:23–9. doi: 10.1016/j.neuint.2013.12.004 24361538

[75] Sies H. Oxidative stress: a concept in redox biology and medicine. Redox Biol. 2015;4:180–3. doi: 10.1016/j.redox.2015.01.002 25588755 PMC4309861

[76] Guo F, Song W, Jiang T, Liu L, Wang F, Zhong H, et al. Electroacupuncture pretreatment inhibits NADPH oxidase-mediated oxidative stress in diabetic mice with cerebral ischemia. Brain Res. 2014;1573:84–91. doi: 10.1016/j.brainres.2014.05.020 24854123

[77] Chen Y, Lei Y, Mo LQ, Li J, Wang MH, Wei JC, et al. Electroacupuncture pretreatment with different waveforms prevents brain injury in rats subjected to cecal ligation and puncture via inhibiting microglial activation, and attenuating inflammation, oxidative stress and apoptosis. Brain Res Bull. 2016;127:248–259. doi: 10.1016/j.brainresbull.2016.10.009 27771396

[78] Shi GX, Wang XR, Yan CQ, He T, Yang JW, Zeng XH, et al. Acupuncture elicits neuroprotective effect by inhibiting NAPDH oxidase-mediated reactive oxygen species production in cerebral ischaemia. Sci Rep. 2015;5:17981. doi: 10.1038/srep17981 26656460 PMC4674709

[79] Wang H, Pan Y, Xue B, Wang X, Zhao F, Jia J, et al. The antioxidative effect of electro-acupuncture in a mouse model of Parkinson’s disease. PLoS One. 2011;6(5):e19790. doi: 10.1371/journal.pone.0019790 21625423 PMC3100295

[80] Koekkoek WA, van Zanten AR. Antioxidant Vitamins and Trace Elements in Critical Illness. Nutr Clin Pract. 2016;31(4):457–74. doi: 10.1177/0884533616653832 27312081

[81] Garrido-Maraver J, Cordero MD, Oropesa-Avila M, Vega AF, de la Mata M, Pavon AD, et al. Clinical applications of coenzyme Q10. Front Biosci (Landmark Ed). 2014;19:619–33. doi: 10.2741/4231 24389208

