# Supplementary figures and images for: The effect of acupuncture on oxidative stress: A systematic review and meta-analysis of animal models
Source: PLoS One. 2022 Sep 9;17(9):e0271098. doi: 10.1371/journal.pone.0271098 (PMC9462787; doi:10.1371/journal.pone.0271098)

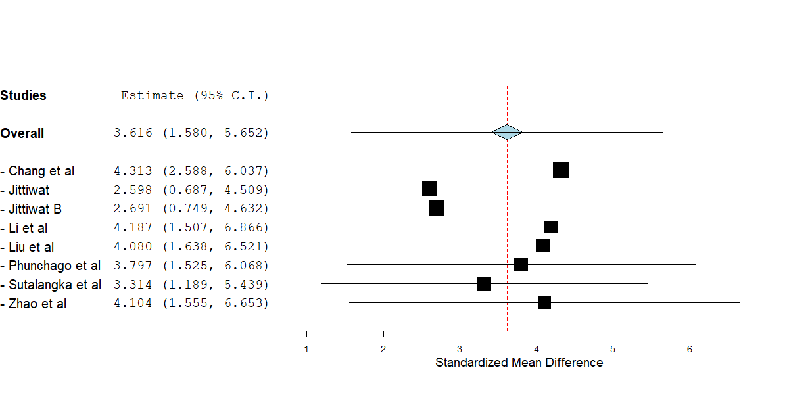

Supplement: S1 Fig — (TIF) [file pone.0271098.s002.tif]

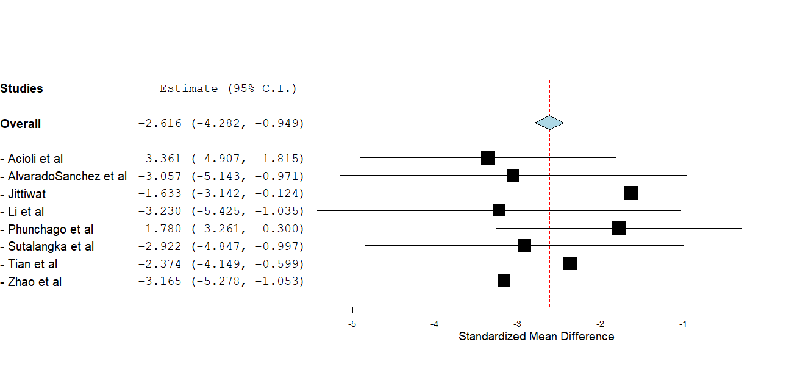

Supplement: S2 Fig — (TIF) [file pone.0271098.s003.tif]

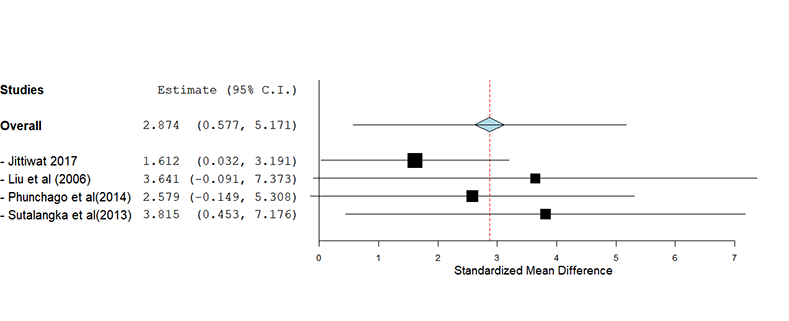

Supplement: S3 Fig — (TIF) [file pone.0271098.s004.tif]

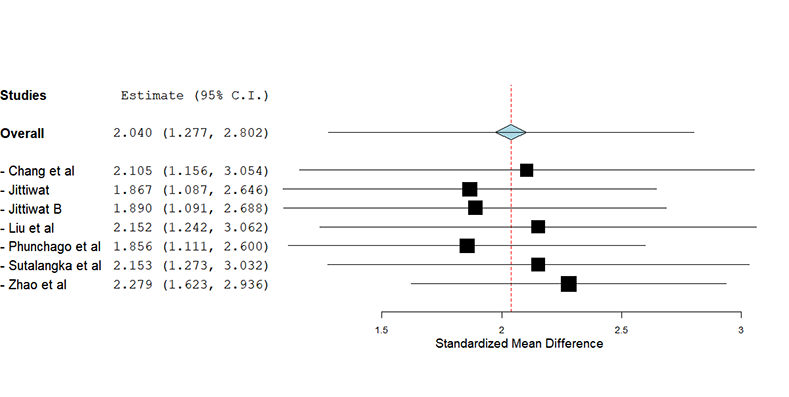

Supplement: S4 Fig — (TIF) [file pone.0271098.s005.tif]
